# The Arabidopsis tonoplast is almost devoid of glycoproteins with complex *N*-glycans, unlike the rat lysosomal membrane

**DOI:** 10.1093/jxb/erv567

**Published:** 2016-01-08

**Authors:** Emanuela Pedrazzini, Andrea Caprera, Ilaria Fojadelli, Alessandra Stella, Alessandra Rocchetti, Barbara Bassin, Enrico Martinoia, Alessandro Vitale

**Affiliations:** ^1^Istituto di Biologia e Biotecnologia Agraria, CNR, Milano, Italy; ^2^Parco Tecnologico Padano, Lodi, Italy; ^3^Institute of Plant Biology, University of Zurich, Zurich, Switzerland

**Keywords:** Asparagine-linked oligosaccharides, cell evolution, glycoproteins, integral membrane proteins, lysosome, plant vacuole, tonoplast.

## Abstract

The distribution of the *N*-glycoproteome in integral membrane proteins of the vacuolar membrane (tonoplast) or the plasma membrane of *Arabidopsis thaliana* and, for further comparison, of the *Rattus norvegicus* lysosomal and plasma membranes, was analyzed. *In silico* analysis showed that potential *N*-glycosylation sites are much less frequent in tonoplast proteins. Biochemical analysis of Arabidopsis subcellular fractions with the lectin concanavalin A, which recognizes mainly unmodified *N*-glycans, or with antiserum against Golgi-modified *N*-glycans confirmed the *in silico* results and showed that, unlike the plant plasma membrane, the tonoplast is almost or totally devoid of *N*-glycoproteins with Golgi-modified glycans. Lysosomes share with vacuoles the hydrolytic functions and the position along the secretory pathway; however, our results indicate that their membranes had a divergent evolution. We propose that protection against the luminal hydrolases that are abundant in inner hydrolytic compartments, which seems to have been achieved in many lysosomal membrane proteins by extensive *N*-glycosylation of the luminal domains, has instead been obtained in the vast majority of tonoplast proteins by limiting the length of such domains.

## Highlight

*N*-Glycosylation is very common in integral membrane proteins of the animal lysosome but it is very rare in Arabidopsis tonoplast proteins, indicating divergent evolution of the lysosomal and vacuolar membranes.

## Introduction

*N*-Glycosylation is a very common modification of proteins synthesized by the secretory pathway. The process is in most cases co-translational and consists of the attachment of the pre-formed oligosaccharide GlcNAc_2_Man_9_Glc_3_ to asparagine residues present in the sequence N-X-S/T (termed sequon), where X cannot be a proline residue. The sequon can be glycosylated only if exposed in the lumen of the endoplasmic reticulum (ER), because the oligosaccharyltransferase is active on the luminal side of the ER translocation channel. Co-translational protein folding can inhibit access of the transferase, further reducing the number of sequons that are actually glycosylated. Data on the presence of oligosaccharide chains in sequons indicate that in mice at least 10% of the total cell proteome is *N*-glycosylated (Zielinska *et al.*, 2010).

Soon after transfer of the oligosaccharide to the polypeptide, the three Glc residues are removed, as part of a cycle of reactions that allow binding and release from ER-located lectins (Helenius and Aebi, 2004). This cycle is involved in the ER-quality control (ERQC) system that promotes productive folding and assembly of secretory proteins and targets structurally defective polypeptides for degradation. Both for this reason and because they directly mask hydrophobic regions of the polypeptide, asparagine-linked oligosaccharides play an important role in allowing correct folding, protection from hydrolytic enzymes, and enhancing the solubility of many proteins (Helenius and Aebi, 2004). It is thus not surprising that *N*-glycosylation is essential for viability in eukaryotes (Ioffe and Stanely, 1994; Yoshida *et al.*, 1995; Koiwa *et al.*, 2003).

When newly synthesized secretory proteins traffic from the ER along the secretory pathway to reach their destination, the *N*-linked glycans can be extensively modified by glycosidases and glycosyltransferases of the Golgi complex (Kornfeld and Kornfeld, 1985; Wilson, 2002; Gomord *et al.*, 2010). The initial modifications are common to all eukaryotes and are necessary for the subsequent, less conserved, steps that give rise to very variable structures collectively termed complex glycans. The attachment of β1,2-linked xylose and α1,3-linked fucose, respectively, to the β-linked mannose residue and the proximal GlcNAc is typical of seed plants, but is present also in a number of lower plant species and has not been found in fungi or vertebrate animals (Gomord *et al.*, 2010). Golgi GlcNAc-transferase I catalyzes one of the initial committed steps of all Golgi modifications and is necessary for embryo development in mice (Ioffe and Stanley, 1994) but not for Arabidopsis development and reproduction (von Schaewen *et al.*, 1993), indicating that the formation of complex glycans has a fundamental role in a model mammal species but not in a higher plant. Consistently, Arabidopsis mutants lacking the Golgi xylosyltransferase and fucosyltransferase activities do not show any defect under normal growth conditions (Strasser *et al.*, 2004). However, upon salt stress, the lack of GlcNAc-transferase I causes a marked decrease in root growth and defects in the biosynthesis of cell wall polysaccharides (Kang *et al.*, 2008). Genetic analysis indicates that the activity of at least one plasma membrane glycoprotein (KOR1/RSW2, which is implicated in cellulose biosynthesis) is negatively affected by the lack of glycan modifications (Kang *et al.*, 2008).

Plant vacuoles are for some important aspects the analogs of animal lysosomes: they share the same position along the secretory pathway and the same role in the degradation of extracellular material as well as intracellular components that are routed to these hydrolytic compartments via endocytic or autophagic events. Other important functions, such as the storage of proteins and secondary metabolites and the role in maintaining the osmotic pressure, are instead specific to plant vacuoles (Marty, 1999).

The tonoplast provides the vacuole with a barrier that separates the vacuolar lumen from the surrounding cytosol and at the same time maintains cytosolic homeostasis through the activities of many integral membrane proteins (Martinoia *et al.*, 2007). However, tonoplast membrane proteins (TMPs), as well as lysosomal membrane proteins (LMPs), are in direct contact with hydrolytic enzymes on the luminal face of the membrane, giving rise to the question of how they are protected from degradation. Many LMPs are highly glycosylated, and most glycans are located in extended loops or terminal domains, probably forming an oligosaccharide coat on the luminal side of the membrane. (Schieweck *et al.*, 2009; Gao *et al.*, 2010). The vacuole as a whole (tonoplast plus soluble content) contains several *N*-glycosylated proteins with a high percentage of complex glycans with β1,2-linked xylose and α1,3-linked fucose, but is devoid of *N*-glycosylated proteins containing the Lewis a epitope (Fitchette *et al.*, 1999). This epitope, also present in mammals, is found in plant plasma membrane and secreted glycoproteins, and is constituted by the terminal structure Galβ(1–3)[Fucα(1–4)]GlcNAc, formed by the action of Golgi glycosyltransferases.

Here we have analyzed the *N*-glycosylation status of Arabidopsis tonoplast proteins, using a combination of biochemical and bioinformatics approaches. Our data show that only few TMPs may contain *N*-linked glycans and even less, or none of these, are of the complex type. Comparison with the Arabidopsis plasma membrane and the rat lysosomal and plasma membranes indicates that this feature is specific for the plant tonoplast. Our observations cast new light on the possible evolution of different strategies to protect membrane proteins of the inner hydrolytic compartments from unwanted proteolysis.

## Materials and methods

### Antibodies

The following antisera and dilutions were used in protein blots: 1:2000 rabbit polyclonal anti-complex glycan (cgly) antiserum (Laurière *et al.*, 1989), 1:1000 chicken polyclonal anti-γTIP antiserum against a synthetic peptide corresponding to the C-terminal nine amino acids of Arabidopsis γ-tonoplast intrinsic protein (TIP) (Rojo *et al.*, 2003), 1:10 000 rabbit polyclonal anti-plasma membrane intrinsic protein 2 (PIP2) antiserum (Santoni *et al.*, 2003), 1:1000 rabbit polyclonal anti-BiP antiserum raised against a recombinant fusion between maltose-binding protein and amino acids 551–667 of tobacco binding protein (BiP) (Pedrazzini *et al.*, 1997), 1:1000 rabbit polyclonal anti-endoplasmin antiserum (Klein *et al.*, 2006), and 1:20 000 peroxidase-conjugated goat anti-rabbit or anti-chicken IgG (Invitrogen). For immunofluorescence microscopy, 1:1000 AlexaFluor 488 goat anti-rabbit (Invitrogen) was used as secondary antibody.

### Plant material

Wild-type or XylT/FucT knockout (Strasser *et al.*, 2004) *Arabidopsis thaliana* plants, ecotype Columbia, were grown in sterile conditions on half-strength Murashige and Skoog (MS) medium (Duchefa Biochemie) supplemented with 10g l^–1^ sucrose and 0.8% (w/v) phyto agar (Duchefa Biochemie) at 23 °C under a 16/8h light/dark cycle. *Arabidopsis thaliana* ecotype Columbia suspension-cultured T87 cells were grown as described (Maîtrejean *et al.*, 2011).

### Isolation of vacuoles from Arabidopsis leaves

Leaves from 4- to 5-week-old plants were scratched on the underside with glass paper (P500) and digested for 90–120min in MCP (0.5M sorbitol, 1mM CaCl_2_, 10mM MES, brought to pH 5.6 using KOH) containing 1% Cellulase R10, 0.5% Macerozyme R10, and 0.04% BSA. The digestion mixes were collected on a 2ml 100% Percoll pH 6 cushion (0.5M sorbitol, 1mM CaCl_2_, 20mM MES, pH 6.0, dissolved in 100% Percoll; GE Healthcare) in a 50ml Falcon tube and centrifuged for 8min, 470 *g*, 20 °C. The supernatant was discarded and a step gradient was then set up as follows (from bottom to top—equal volumes of each step): (i) protoplasts in 40% Percoll pH 6; (ii) 30% Percoll pH 7.2 (0.5M sorbitol, 20mM HEPES dissolved in 30% Percoll, pH 7.2); and (iii) sorbitol buffer (0.4M sorbitol, 30mM K-gluconate, 20mM HEPES, pH 7.2 with imidazole). After centrifugation at 300 *g*, 8min, 20 °C, protoplasts were recovered at the 30% Percoll/sorbitol buffer interface and transferred into a 50ml Falcon tube. Lysis was performed by mixing with an equal volume of lysis buffer (0.2M sorbitol, 20mM EDTA, 10mM HEPES, 10% Ficoll, pH 8.0, 1mM DTT, 0.016% BSA, pre-warmed to 42 °C). Lysis was stopped after 10min by placing the tube on ice. The released vacuoles were purified using a second step gradient set up as follows (from bottom to top): (i) 5ml of lysate; (ii) 5ml of 1:1 lysis buffer:betaine buffer (0.4M betaine, 30mM K-gluconate, 1mM DTT, 0.1% BSA, 20mM HEPES, brought to pH 7.2 using 1M imidazole) supplemented with 10% BSA, 1M DTT; and (iii) betaine buffer. After centrifugation at 1500 *g*, 8min, 20 °C, vacuoles were recovered at the interface between the (ii) and (iii) cushions. Vacuoles were frozen in liquid N_2_ and stored at –70 °C.

### Tonoplast and total microsome purification

Purified vacuoles or intact protoplasts, containing the same amount of α-mannosidase activity, measured as described (Maîtrejean *et al.*, 2011), were freeze-thawed three times, vigorously mixed by pipetting, and centrifuged at 2500 *g* for 10min at 4 °C. Recovered supernatants were placed in 0.5M sorbitol, 20mM HEPES pH 7.2, Complete protease inhibitor cocktail (Roche), and incubated for 20min in ice. To strip peripheral membrane proteins, supernatants were further treated with 0.1M Na_2_CO_3_. To enrich tonoplast or total microsomes, the samples were centrifuged for 1h, 100 000 *g*, 4 °C. Supernatants, containing vacuole or protoplast soluble fractions were recovered. Membrane pellets, containing tonoplast or total microsomes, were resuspended in 0.5M sorbitol, 20mM HEPES pH 7.2, using 1:10 volume with respect to the soluble fractions. An equal volume, or equal amount of proteins, of soluble or membrane fractions was analyzed by SDS–PAGE and protein blot on a nitrocellulose membrane (Perkin-Elmer), and incubated with appropriate antibodies and anti-rabbit or anti-chicken IgG–peroxidase conjugate (Pierce) For detection of proteins with high-mannose *N*-glycans, the protein blot was incubated with 3 µg ml^–1^ concanavalin A (ConA)–peroxidase conjugate (Sigma-Aldrich St. Louis, MO, USA) in phosphate-buffered saline (PBS) containing 0.05% (v/v) Tween-20, 1mM CaCl_2_, 1mM MnCl_2_, and 1mM MgCl_2_ for 16h at 20 °C, according to the manufacturer’s protocols. Peroxidase activity can be detected using Super West Pico (Pierce) according to the manufacturer’s protocol. Protein molecular weight markers (Fermentas) were used as SDS–PAGE molecular mass markers.

### Analysis of total leaf proteins

For total protein extraction from wild-type or XylT/FucT knockout plants, Arabidopsis leaves (3–6 weeks old) were homogenized in ice-cold homogenization buffer (200mM NaCl, 1mM EDTA, 0.2% Triton X-100, 2% 2-mercaptoethanol, 100mM TRIS-Cl pH 7.8) supplemented with Complete protease inhibitor cocktail. After centrifugation at 5000 *g*, 10min, 4 °C, the resulting supernatant was considered as the total protein extract. Proteins were analyzed by SDS–PAGE and protein blot.

### Microsome subfractionation

Microsome subfractionation was performed according to Ishikawa *et al.* (2005). Total microsomes were prepared as follows: *A. thaliana* leaf tissue was homogenized in a buffer containing 50mM TRIS-acetate (pH 7.5), 250mM sorbitol, 2mM EGTA, 2mM MgCl_2_, 2mM DTT supplemented with Complete protease inhibitor cocktail. The homogenate was filtered and centrifuged at 10 000 *g* for 10min at 4 °C. The supernatant (S10) was further centrifuged at 100 000 *g* (rav) in a Beckman SW55Ti rotor (Beckman Instruments) for 2h at 4 °C. The resulting pellet (P100), containing total microsomes, was resuspended in 10mM Tricine-KOH (pH 7.5), 1mM EGTA, 2mM MgCl_2_, 5% (w/w) sucrose, and was loaded on a sucrose density gradient [10.4ml, 15–45% (w/w) sucrose in 10mM Tricine-KOH (pH 7.5), 1mM EGTA, and 2mM MgCl_2_] and centrifuged at 77 000 *g* (rav) for 19h in a Beckman SW40 rotor. Twenty fractions of 0.55ml were collected. The precipitate at the bottom of the tube was solubilized with 550 μl of SDS–PAGE loading buffer. Equal volumes of each fraction and of the solubilized precipitate were analyzed by SDS–PAGE and protein blot.

### Immunofluorescence of protoplasts from Arabidopsis cell culture

Protoplasts were prepared from 7-day-old cell culture as described (Maîtrejean *et al.*, 2011). After the last wash in W5 buffer, protoplasts were fixed by resuspension in MaCa buffer [0.5M mannitol, 20mM CaCl_2_, and 0.1% (w/v) MES, pH 5.7] containing 4% (w/v) paraformaldehyde, for 2h at 20 °C. Immunofluorescence was performed as described (Maggio *et al.*, 2007). After immunolabeling, protoplasts were resuspended in ProLong Gold antifade reagent (Invitrogen-Molecular Probes, Eugene, OR, USA) and visualized by epifluorescence microscopy using a Zeiss Axiovert 200 microscope (Carl-Zeiss, Oberkochen, Germany), followed by the collection of optical sections using the Zeiss Apotome system and Axiovision 4.1 software. Figures were assembled using Adobe Photoshop software (Adobe Systems Incorporated, CA, USA).

### *In silico* analysis

The goal of the entire procedure (pipeline) is the *in silico* identification of putative *N*-glycosylation signals in proteins already known to be localized on lysosomal, vacuolar, or plasma membranes. The procedure starts with the analysis of proteins identified by their TAIR or IPI IDs. Uniprot identifiers are also used to obtain information from external databases (e.g. Fasta formatted sequences or protein annotations). Conversions among identifiers are made through ‘programmatic’ access to the Uniprot database (http://www.uniprot.org/faq/28) using Ruby programming language scripts (https://www.ruby-lang.org/en/) or, as an alternative, using the Uniprot Mapping facility (http://www.uniprot.org/mapping/). The analysis procedure includes searching for *N*-glycosylation signals and the investigation of putative transmembrane regions and related sequence topology. Home-made scripts, in the Ruby programming language, have been written in order to automate the entire procedure. These scripts are used to link together successive steps of the bioinformatics analysis (pipeline) and guarantee compatibility of data formats between different steps. Protein annotations and data obtained during the analysis are progressively stored in a local database. An internal SQLite database (http://www.sqlite.org/) has been created. The Database Management System is used for data access and querying by means of the SQL language (http://en.wikipedia.org/wiki/SQL). Putative transmembrane regions and sequence membrane topology are investigated using local installations of two alternative tools, for cross-validation: TMHMM (http://www.cbs.dtu.dk/services/TMHMM/) and Phobius (http://phobius.sbc.su.se/). Sequons are searched by means of a local installation of the NetNGlyc software (http://www.cbs.dtu.dk/services/NetNGlyc/). The algorithm indicates as putative *N*-glycosylation sites only signals localized in the extracytoplasmic side(s) of the transmembrane proteins, and positioned at minimal distances of 12 and 14 residues, after the end and before the beginning of a transmembrane segment, respectively.

## Results

### *In silico* analysis suggests that *N*-glycosylated proteins are under-represented in the Arabidopsis tonoplast proteome

To obtain an overview on the putative *N*-glycosylation status of Arabidopsis membrane proteins, *in silico* analysis was performed, searching for *N*-glycosylated membrane proteins and their subcellular distribution. Published proteomics data on the Arabidopsis vacuole (tonoplast plus soluble proteins) or tonoplast (Carter *et al.*, 2004; Sazuka *et al.*, 2004; Shimaoka *et al.*, 2004; Szponarski *et al.*, 2004; Jaquinod *et al.*, 2007) were first compared. Ninety-five sequences that had been assigned to the tonoplast in all these studies were selected. To enrich our TMP database, a search for proteins annotated as ‘tonoplast’ was performed in the TAIR database; integral membrane proteins were extracted from the resulting list using the ‘bulk protein search’ tool and, if not already present, they were added to our database (Supplementary Table S1 at *JXB* online, A.th.TMPs, highlighted in blue). Finally, proteins for which there is published experimental evidence for a localization other than the tonoplast were discarded. In total, we thus identified 109 integral tonoplast proteins (Supplementary Table S1, A.th.TMPs).

A search and selection using the same criteria was performed on Arabidopsis plasma membrane proteins (PMPs). Proteomic analysis by Alexandersson *et al.* (2004) reported 238 putative PMPs. This study was chosen because it contains the highest number of identified polypeptides. Integral membrane proteins annotated as ‘Plasma membrane’ were also extracted from the TAIR protein database. When the two lists were combined, proteins that were experimentally verified to be not at the plasma membrane were discarded and only integral membrane proteins were considered; 183 proteins were thus selected (Supplementary Table S2, A.th.PMPs).

Putative sequon positions in the selected proteins were predicted by a bioinformatics procedure (pipeline) that combined the analysis of transmembrane protein topology by the TMHMM or Phobius software and the search for putative *N*-glycosylation sites by the NetNGlyc software (tripeptides with proline in the X position were excluded). The following further limitations were considered. Amino acids located in cytosolic or transmembrane regions cannot come into contact with the oligosaccharyltransferase and therefore cannot be glycosylated. Moreover, close proximity to transmembrane domains or the N-terminal signal peptide inhibits glycosylation, because of steric effects (Nilsson and von Heijne, 1993; Chen *et al.*, 2001): mutagenesis indicated that, to be glycosylated, the luminal sequon must be at minimal distances of 12 and 14 residues after the end and before the beginning of a transmembrane segment, respectively (Nilsson and von Heijne, 1993; Cheung and Reithmeier, 2007). Consistently, the single luminal sequon of the tonoplast tandem-pore potassium channel 1 (TPK1, At5g55630) that immediately precedes the second transmembrane helix is not glycosylated in vivo (Maîtrejean *et al.*, 2011). *N*-Glycosylation sites that did not meet these requirements were excluded.

Analysis using the TMHMM algorithm indicated that only 17 out of the 109 TMPs can be glycosylated, representing 15.6% of the tonoplast proteome and having in total 41 sequons (Table 1; for details see Supplementary Table S1, TMHMM_A.th.TMPs N-glyc sites and TMHMM_N-glyc A.th.TMPs). The Phobius algorithm was more selective: 12 sequences were validated, with 28 sequons (Supplementary Table S1, Phobius_A.th.TMPs N-glyc sites and Phobius_ N-glyc A.th.TMPs; note that 11 sequences were shared by both algorithms).

**Table 1.** Putative *N*-glycosylated integral tonoplast proteins (N-glyc TMPs) of *Arabidopsis thaliana*, identified using NetGlyc and TMHMM algorithms

**TAIR ID**	**No. of sequons**	**Description**
AT1G75630.2	1	V-type proton ATPase 16kDa proteolipid subunit c4
AT3G51490.1	1	Monosaccharide-sensing protein 3 (Sugar transporter MSSP3)
AT5G14120.1	1	Major facilitator protein (Nodulin-like protein)
AT5G62890.1	1	Nucleobase-ascorbate transporter 6 (AtNAT6)
AT2G28520.1	2	Vacuolar proton ATPase a1 (95kDa subunit a isoform 1)
AT2G34660.1	2	ABC transporter C family member 2 (ABC transporter AtABCC2)
AT2G38170.1	2	Vacuolar cation/proton exchanger 1 (Ca(2+)/H(+) antiporter CAX1)
AT2G47600.1	2	Magnesium/proton exchanger (Mg(2+)/H(+) exchanger) (AtMHX)
AT3G03720.1	2	Cationic amino acid transporter 4
AT3G62700.1	2	ABC transporter C family member 14 (ABC transporter AtABCC14)
AT4G01840.1	2	Two-pore potassium channel 5 (AtTPK5)
AT5G40890.1	2	Chloride channel protein CLC-a (AtCLC-a)
AT5G46370.1	2	Two-pore potassium channel 2 (AtTPK2)
AT5G61350.1	2	Protein kinase superfamily protein
AT5G39040.1	3	ABC transporter B family member 27 (ABC transporter AtABCB27) (Aluminum tolerance-related ATP-binding cassette transporter AtTAP2)
AT5G45890.1	6	Senescence-associated gene 12
AT5G48410.1	8	Glutamate receptor 1.3

Among the 183 integral proteins of the plasma membrane, 82 polypeptides, representing 45% of the total, contain 370 sequons that satisfy the criteria described above for *N*-glycosylation when analyzed by the TMHMM algorithm (Table 2; for details see Supplementary Table S2, TMHMM_A.th. PMPs N-glyc sites and TMHMM_N-glyc A.th.PMPs). Also in this case the Phobius algorithm is more stringent, identifying 71 putative *N*-glycosylated PMPs in Arabidopsis for a total of 319 sequons (Supplementary Table S2, Phobius_A.th.PMPs N-glyc sites and Phobius_N-glyc A.th.PMPs, for more details).

**Table 2.** Putative N-glycosylated integral plasma membrane proteins (N-glyc PMPs) of *Arabidopsis thaliana*, identified using NetGlyc and TMHMM algorithms

**TAIR_ID**	**No. of sequons**	**Description**
AT1G08700.1	1	PS1, Presenilin-1
AT1G15080.1	1	ATPAP2, lipid phosphate phosphatase 2
AT1G17620.1	1	Late embryogenesis abundant (LEA) hydroxyproline-rich glycoprotein family
AT1G52200.1	1	Protein PLANT CADMIUM RESISTANCE 8 (AtPCR8)
AT2G03620.1	1	Magnesium transporter MRS2-5 (Magnesium Transporter 3, AtMGT3)
AT3G28007.1	1	SWEET4, Nodulin MtN3 family protein
AT3G28450.1	1	Leucine-rich repeat protein kinase-like protein
AT3G45600.1	1	Tetraspanin-3
AT3G46900.1	1	COPT2, copper transporter 2
AT3G62360.1	1	Carbohydrate-binding-like fold-containing protein
AT4G29870.1	1	Oligosaccharyltransferase complex/magnesium transporter family protein
AT5G07390.1	1	ATRBOHA, respiratory burst oxidase homolog A
AT5G35390.1	1	Leucine-rich repeat protein kinase family protein
AT5G59030.1	1	Copper transporter 1 (AtCOPT1)
AT5G64080.1	1	Bifunctional inhibitor/lipid-transfer protein/seed storage 2S albumin superfamily protein
AT1G59870.1	2	ABC transporter G family member 36 (ABC transporter AtABCG36)
AT2G27500.1	2	Glycosyl hydrolase superfamily protein
AT2G36850.1	2	ATGSL08, glucan synthase-like 8
AT3G09740.1	2	Syntaxin-71 (AtSYP71)
AT3G28860.1	2	ABC transporter B family member 19 (ABC transporter AtABCB19)
AT3G54200.1	2	Late embryogenesis abundant hydroxyproline-rich glycoprotein
AT4G04970.1	2	ATGSL1, glucan synthase-like 1
AT4G13510.1	2	Ammonium transporter 1 member 1 (AtAMT1)
AT4G30190.2	2	ATPase 2
AT5G14870.1	2	CNGC18, cyclic nucleotide-gated channel 18
AT5G25090.1	2	ENODL13, early nodulin-like protein 13
AT1G05570.1	3	CALS1, callose synthase 1
AT1G13110.1	3	Cytochrome P450 71B7
AT1G17840.1	3	WBC11, white-brown complex homolog protein 11
AT1G32860.1	3	Glycosyl hydrolase superfamily protein
AT1G75680.1	3	Endoglucanase 10
AT3G26700.1	3	Putative uncharacterized protein
AT3G51050.1	3	FG-GAP repeat-containing protein
AT3G59100.1	3	ATGSL11, glucan synthase-like 11
AT4G03550.1	3	Callose synthase 12
AT4G28100.1	3	Unknown protein
AT5G11420.1	3	Putative uncharacterized protein
AT5G11560.1	3	Putative uncharacterized protein
AT5G19230.1	3	Glycoprotein membrane precursor GPI-anchored
AT1G21880.2	4	LYM1, lysm domain GPI-anchored protein 1 precursor
AT1G65240.1	4	Eukaryotic aspartyl protease family protein
AT3G02880.1	4	Probable inactive receptor kinase
AT3G07160.1	4	Callose synthase 9
AT3G23750.1	4	BAK1-ASSOCIATING RECEPTOR-LIKE KINASE 1
AT4G18760.1	4	Putative uncharacterized protein
AT4G31140.1	4	*O*-Glycosyl hydrolases family 17 protein
AT5G06320.1	4	Harpin-induced protein-like (NDR1/HIN1-Like protein 3)
AT5G13000.1	4	ATGSL12, glucan synthase-like 12
AT5G28680.1	4	ANX2, Malectin/receptor-like protein kinase family protein
AT5G51060.1	4	RHD2, NADPH/respiratory burst oxidase protein D
AT5G67130.1	4	PLC-like phosphodiesterases superfamily protein
AT1G06490.1	5	GSL7, glucan synthase-like 7
AT3G04690.1	5	ANX1, Malectin/receptor-like protein kinase family protein
AT4G29360.1	5	*O*-Glycosyl hydrolases family 17 protein
AT5G19250.1	5	Glycoprotein membrane precursor GPI-anchored
AT3G13560.1	6	*O*-Glycosyl hydrolases family 17 protein
AT3G14570.1	6	ATGSL04, glucan synthase-like 4
AT3G20600.1	6	NDR1, Late embryogenesis abundant (LEA) hydroxyproline-rich glycoprotein family
AT3G46550.1	6	SOS5, Fasciclin-like arabinogalactan family protein
AT4G23950.2	6	Galactose-binding protein
AT5G36870.1	6	ATGSL09, glucan synthase-like 9
AT5G49720.1	6	ATGH9A1, KOR1, glycosyl hydrolase 9A1
AT5G58480.1	6	*O*-Glycosyl hydrolases family 17 protein
AT1G66970.2	7	Probable glycerophosphoryl diester phosphodiesterase 3
AT2G13680.1	7	CALS5, callose synthase 5
AT2G17120.1	7	LYM2, lysm domain GPI-anchored protein 2 precursor
AT2G37710.1	7	L-type lectin-domain containing receptor kinase IV.1
AT2G26730.1	8	Probable inactive receptor kinase
AT2G31960.1	8	ATGSL03, glucan synthase-like 3
AT3G51550.1	8	Receptor-like protein kinase FERONIA
AT5G38990.1	8	Probable receptor-like protein kinase
AT5G49760.1	8	Leucine-rich repeat protein kinase-like protein
AT5G55480.1	8	SVL1, SHV3-like 1
AT3G29810.1	9	COBRA-like protein 2 precursor
AT4G25240.1	9	SKS1, SKU5 similar 1
AT5G49150.1	9	ATGEX2, gamete expressed 2
AT5G51480.1	9	SKS2, SKU5 similar 2
AT4G39400.1	10	BRI1, Leucine-rich receptor-like protein kinase family protein
AT1G74790.1	11	Unknown protein
AT1G53430.1	13	Probable LRR receptor-like serine/threonine-protein kinase
AT3G14840.2	14	Probable leucine-rich repeat receptor-like serine/threonine-protein kinase
AT4G08850.1	18	Probable LRR receptor-like serine/threonine-protein kinase

**Fig. 1.**
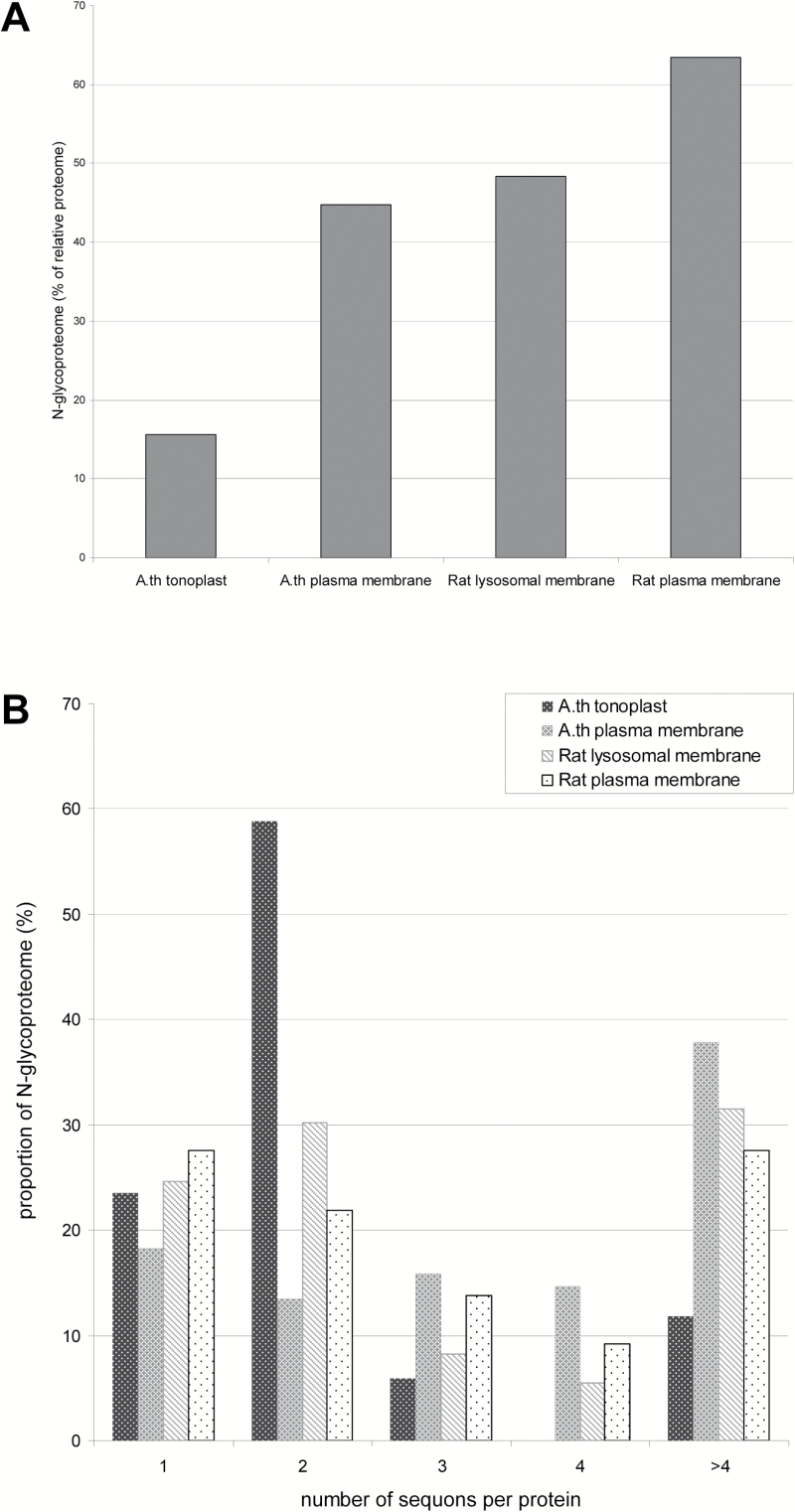
The *N*-glycoproteome is under-represented in the Arabidopsis tonoplast. (A) Percentage of polypeptides containing sequons that satisfy the requirements for *N*-glycosylation (*N*-glycoproteome) in the proteomes of the Arabidopsis tonoplast or plasma membrane, and of the rat lysosomal or plasma membranes. (B) Distribution of sequons per protein in the four *N*-glycoproteomes illustrated in (A). The TMHMM algorithm was used to define transmembrane domains and protein topology.

These data indicate that a much lower fraction of the tonoplast than the plasma membrane proteome may be *N*-glycosylated (Fig. 1A) and that glycoproteins with more than two sequons are rare in the tonoplast but very frequent in the plasma membrane (Fig. 1B). This suggests an enrichment of *N*-glycans on the cell surface and a selective depletion on the tonoplast.

### The Arabidopsis tonoplast has a much smaller proportion of putative *N*-glycosylated proteins than the rat lysosomal membrane

As mentioned in the Introduction, vacuoles and lysosomes share a number of features and functions. We thus wondered whether the observations we made regarding Arabidopsis can be extended to a model animal. The recently described rat (*Rattus norvegicus*) liver lysosomal proteome (Chapel *et al.*, 2013) was first analyzed, searching for putative LMPs. We thus excluded the proteins with ‘detailed localization’ C, cytoplasm; CS, cytoskeleton; ER, endoplasmic reticulum; G, Golgi; EG: endoplasmic reticulum, Golgi; M, mitochondria; P, peroxisomes; N, nucleus; MPN: mitochondria, peroxisomes, nucleus; PM, plasma membrane (see table S4b in Chapel *et al.*, 2013). The remaining 151 sequences (Supplementary Table S3, ratLMPs) were then scanned through our pipeline in order to identify *N*-glycosylated LMPs.

Seventy-three putative LMPs that can be *N*-glycosylated were identified for a total of 291 sequons (Supplementary Table S3, TMHMM_ratLMPs N-glyc sites and TMHMM_N-glyc ratLMPs); these represent 48% of the entire lysosomal membrane proteome. Using the Phobius algorithm, 75 *N*-glycosylated LMPs were identified, for a total of 275 sequons (Supplementary Table S3, Phobius_ratLMPs N-glyc sites and Phobius_N-glyc ratLMPs). It can be concluded that the relative abundance of putative *N*-glycoproteins in the lysosomal membrane is three times greater than in the tonoplast (Fig. 1A). In this respect, the lysosomal membrane resembles the Arabidopsis plasma membrane more than the tonoplast, a feature confirmed when the distribution of sequons per glycoprotein is analyzed (Fig. 1B). These results mark a major, perhaps unsuspected, difference between the vacuolar and lysosomal membranes.

We then verified whether there is any marked difference in the abundance of *N*-glycosylation between the lysosomal and plasma membrane proteomes in rat liver cells. Proteomic analysis by Zhou *et al.* (2011) listed 950 PMPs (Supplemental table 1A ‘Proteins identified from plasma membrane’ in Zhou *et al.*, 2011), among which 465 have at least one putative transmembrane domain. We have further refined the list of putative integral PMPs using the PANTHER gene ontology classification system; we have selected genes that are classified as ‘plasma membrane’ and excluded those classified as ‘ER’, ‘mitochondria’, or ‘lysosome’. This analysis restricted the number of rat PMPs to 137, here reported in Supplementary Table S4 (ratPMPs). Among this group of proteins, 87 can be *N*-glycosylated for a total of 296 sequons, identified using the TMHMM algorithm (Supplementary Table S4, TMHMM_ratPMPs N-glyc sites and TMHMM_N-glyc ratPMPs), thus representing 63% of the total. When the Phobius algorithm was used, 81 putative *N*-glycosylated proteins with 277 sequons were identified (Supplementary Table S4, Phobius_ratPMPs N-glyc sites and Phobius_N-glyc ratPMPs).

The ratio between the percentages of putative *N*-glycosylated proteins in the rat plasma membrane and the lysosomal membrane is therefore 1.31 (63%/48%, Fig. 1A) compared with the 2.88 ratio (45%/15.6%) between the Arabidopsis plasma membrane and tonoplast. The distribution of the number of sequons per polypeptide confirms that there is not a marked difference in *N*-glycosylation patterns between the two rat membranes, unlike what we observed in Arabidopsis (Fig. 1B). Therefore, the low presence of *N*-glycosylation sites in the membrane proteins of the hydrolytic subcellular compartment relative to those of the plasma membrane is a feature of the model plant, not reflected in the model animal.

### Biochemical characterization of the *N*-glycoproteome distribution in Arabidopsis

To obtain further experimental support for the bioinformatics results, biochemical analysis of the *N*-glycosylation status of proteins in the different Arabidopsis membranes was performed.

**Fig. 2.**
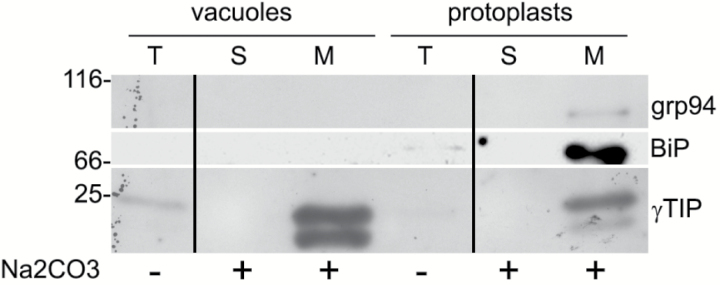
The tonoplast preparation is not contaminated by ER microsomes. Protoplasts from Arabidopsis leaves were used to isolate vacuoles. The isolated vacuoles or protoplast preparations (T) were then separated into microsomal (M) and soluble (S) fractions, in the presence (+) or absence (–) of Na_2_CO_3_. Equal amounts of protein were analyzed by SDS–PAGE and protein blot, using antisera against endoplasmin/grp94 (grp94), BiP, or γTIP. The vertical lines separate lanes rearranged from different parts of a single blot exposure. Numbers on the left indicate the positions of molecular mass markers, in kDa.

**Fig. 3.**
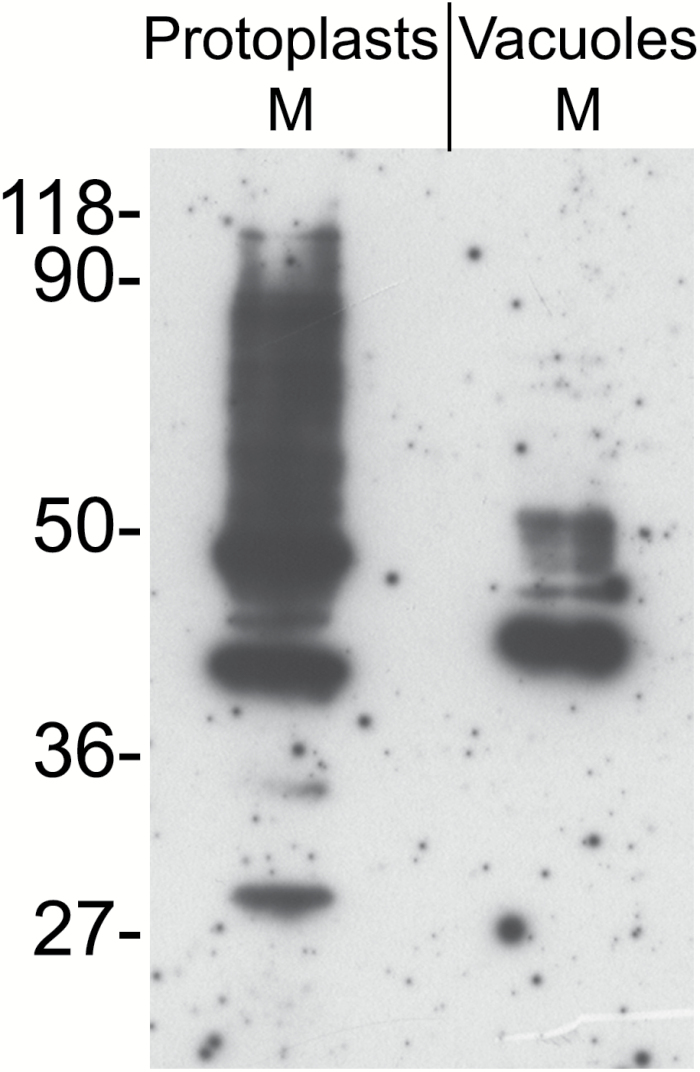
*N*-Glycosylated proteins with high-mannose oligosaccharide chains are much less abundant in the tonoplast than in the total membrane fraction. Microsomal fractions (M) from Arabidopsis protoplasts or purified vacuoles were analyzed by SDS–PAGE and protein blot followed by incubation with concanavalin A conjugated to peroxidase. Numbers on the left indicate the positions of molecular mass markers, in kDa.

**Fig. 4.**
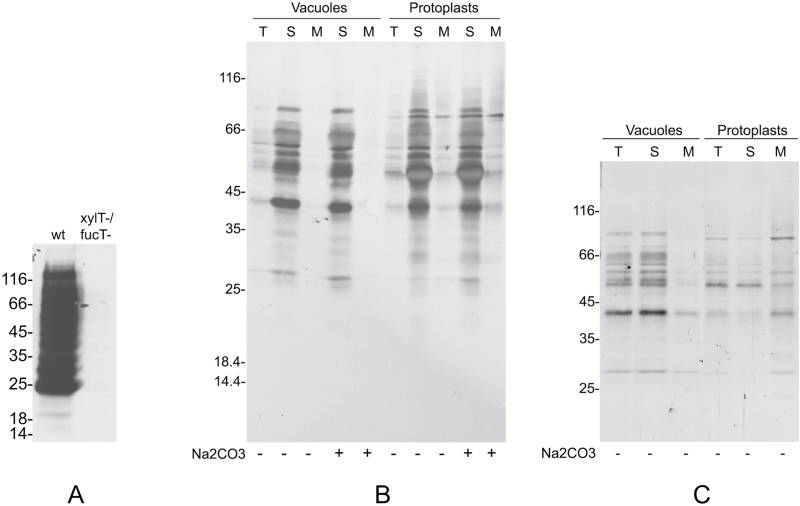
The cgly serum does not detect polypeptides in the tonoplast preparation. In each panel, analysis was by SDS–PAGE and protein blot using anti-cgly serum. (A) Total proteins were extracted from leaves of wild-type (wt) or xylT–/fucT– Arabidopsis. Equal amounts of protein were analyzed. (B) Soluble (S) and microsomal (M) fractions were prepared from vacuoles or protoplasts in the presence (+) or absence (–) of Na_2_CO_3_. An equal proportion of each fraction, or a 10-fold dilution of each total unfractionated sample (T), was analyzed. (C) As in (B) but an equal amount of protein was analyzed for each fraction. In each panel, numbers on the left indicate the positions of molecular mass markers, in kDa.

Vacuoles were isolated from protoplasts released by digestion of Arabidopsis rosette leaves. Microsomes were then prepared either from the purified vacuolar fraction or from whole protoplasts and were treated with Na_2_CO_3_ to release peripheral membrane proteins. Microsomes from the vacuolar fraction should thus be constituted by the tonoplast, whereas those from whole protoplasts should contain all membranes of the cell. Protein blots using antibodies against the tonoplast marker γ-TIP (Rojo *et al.*, 2003) or the ER markers BiP (Pedrazzini *et al.*, 1997) and endoplasmin/grp94 (Klein *et al.*, 2006) showed that in the Na_2_CO_3_-stripped vacuolar microsomes γ-TIP was highly enriched, whereas the ER markers were below our limit of detection (Fig. 2). Conversely, the two ER residents were clearly detected in the protoplast microsomal preparation. The tonoplast fraction was therefore only negligibly contaminated by membranes originating from the major compartment of the endomembrane system. To detect the occurrence of *N*-glycans on TMPs, protein blot was performed by incubation with ConA conjugated to peroxidase. ConA binds terminal α-d-mannosyl and α-d-glucosyl residues, mainly occurring in high-mannose, unmodified *N*-glycans (Goldstein and Poretz, 1986). Very few polypeptides were recognized by ConA in the tonoplast compared with those recognized in total microsomes (Fig. 3). This may indicate that very few TMPs are *N*-glycosylated or that the vast majority of TMP *N*-glycans are modified during traffic through the Golgi apparatus. To investigate these two possibilities, tonoplast microsomes were tested for the presence of modified *N*-glycans (complex glycans, cgly), using an anti-cgly antiserum specific for β1,2-linked xylose, a residue present in all cgly detectable in the Arabidopsis proteome (Faye and Chrispeels, 1988; Laurière *et al.*, 1989; Strasser *et al.*, 2004). Consistently, protein blot of Arabidopsis total leaf homogenates showed that no polypeptide was recognized by anti-cgly in knockout plants totally deficient in Golgi xylosyl- and fucosyltransferase activities (xylT–/fucT–) (Strasser *et al.*, 2004), but many polypeptides were detected in wild-type plant extracts (Fig. 4A). The antiserum was therefore used to challenge subcellular fractions of wild-type protoplasts and vacuoles (Fig. 4B; note that soluble and microsomal fractions derived from the same number of cells were analyzed). In both protoplasts and vacuoles, the vast majority of polypeptides recognized by the anti-cgly antiserum were in the soluble fraction, strongly suggesting that most intracellular glycoproteins with cgly are soluble proteins of the vacuolar lumen. A small number of microsomal proteins from protoplasts also reacted with the antiserum. Some of these were possibly minor contaminations from abundant soluble proteins, but others were relatively enriched in the Na_2_CO_3_-stripped microsomes. The patterns were very similar when microsomes were not stripped. Microsomes isolated from vacuoles did not give any signal, indicating that cgly are either absent or present in a very minor proportion in the tonoplast proteome. Even when an equal amount of protein was analyzed for each subcellular fraction and membranes were not stripped, the signal given by the tonoplast preparation was very low compared with that of protoplast microsomes (Fig. 4C; the residual bands very probably reflect contaminations from soluble proteins, since no enrichment of a specific band was detectable).

Taken together, the ConA-binding and anti-cgly assays confirm our *in silico* results, indicating a low frequency of *N*-glycosylated proteins in the tonoplast proteome.

### Most Arabidopsis membrane proteins with complex glycans are at the plasma membrane

**Fig. 5.**
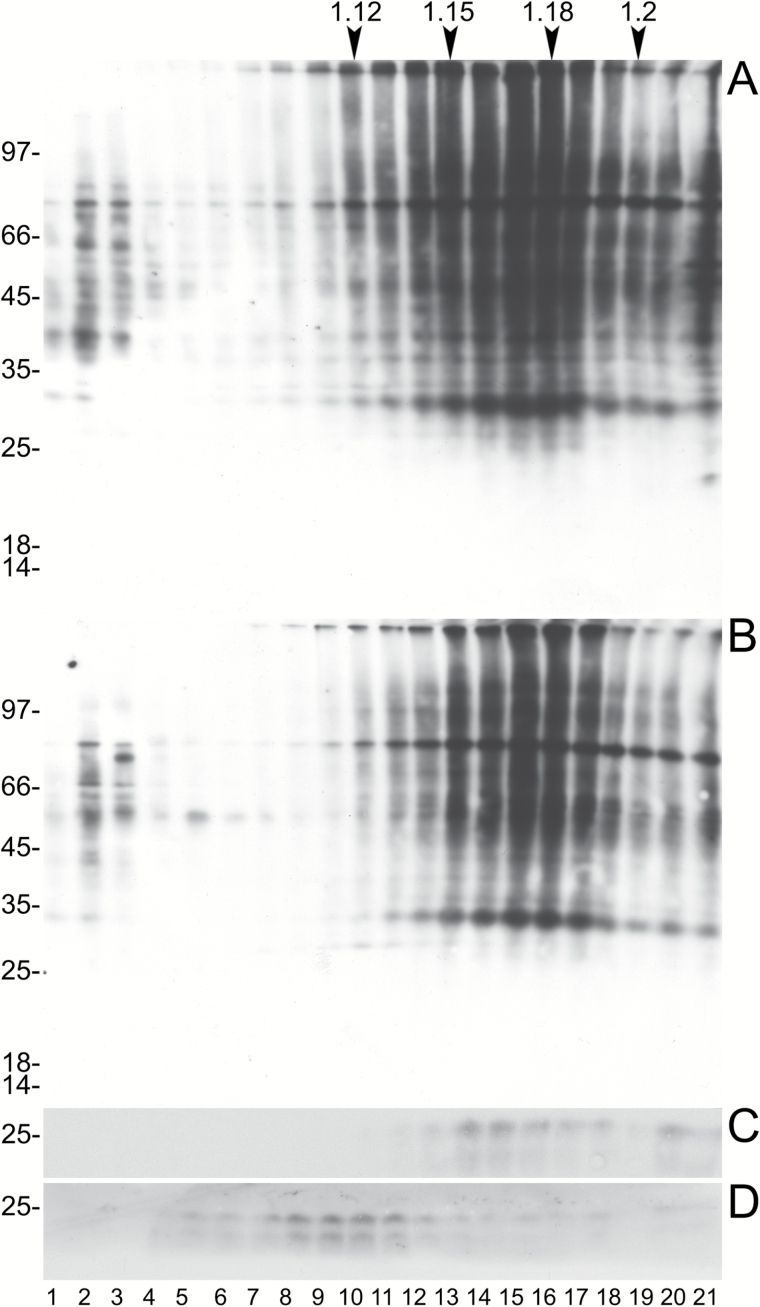
Most membrane proteins with complex glycans are in a microsomal fraction with higher density than the tonoplast. Microsomes were prepared from Arabidopsis leaves in the absence (A, C, D) or presence (B) of Na_2_CO_3_ and subjected to isopycnic sucrose gradient centrifugation. Analysis of each gradient fraction was by SDS–PAGE and protein blot using anti-cgly (A, B), anti-PIP (C), or anti-γTIP (D) sera. Numbers at the top indicate the fraction density. In each panel, numbers on the left indicate the positions of molecular mass markers, in kDa.

**Fig. 6.**
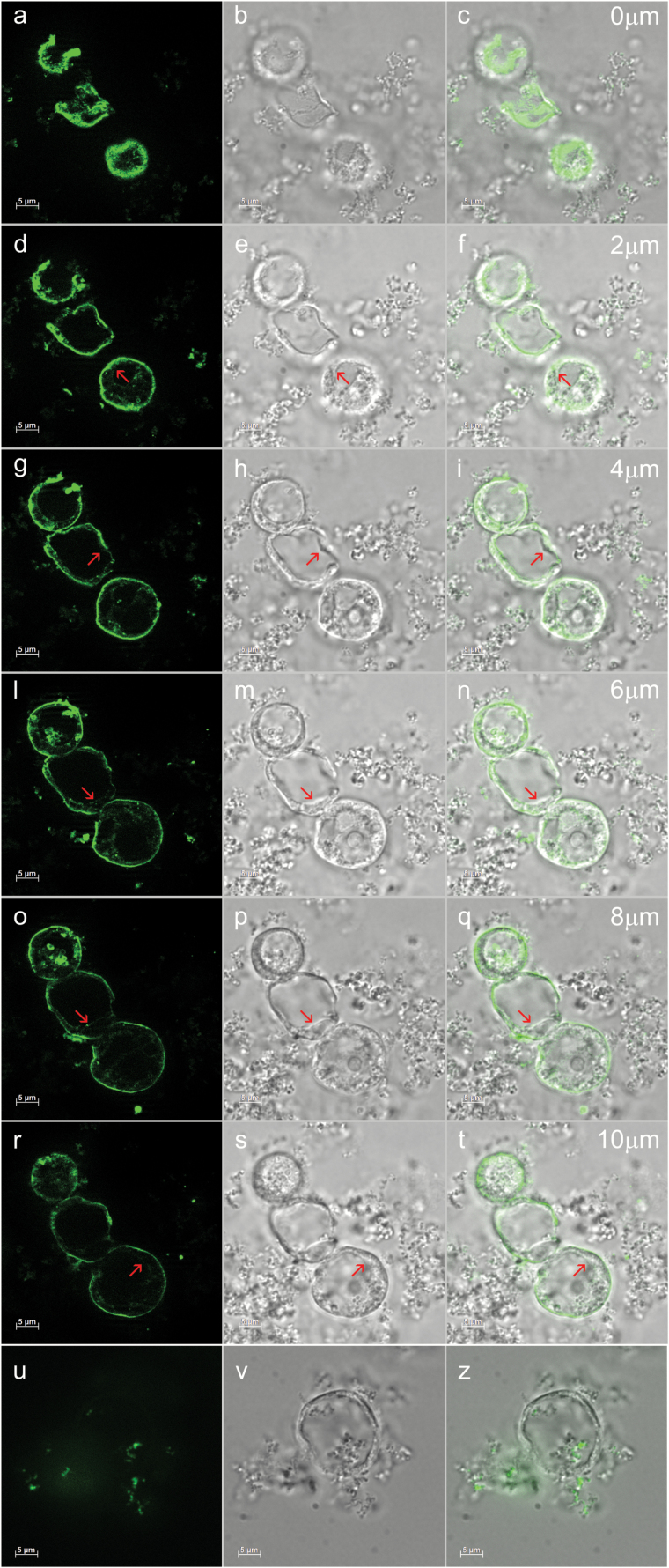
The majority of membrane proteins with complex glycans are in the plasma membrane. (A) Protoplasts isolated from Arabidopsis cultured cells were fixed, permeabilized, and analyzed by immunofluorescence microscopy using anti-cgly antibody and AlexaFluor 488 goat anti-rabbit secondary antibody. As control, the anti-cgly antibody was omitted and incubation was only performed with AlexaFluor 488 goat anti-rabbit secondary antibody (u, z). Six *Z*-stacks (thickness: 2 µm) were collected using the Apotome system. a, d, g, l, o, r, anti-cgly/AlexaFluor 488 anti-rabbit IgG; u, AlexaFluor 488 goat anti-rabbit IgG; b, e, h, m, p, s, v, brightfield; c, f, i, n, q, t, z, merge. Red arrows point to the tonoplast. Scale bars=5 µm.

To obtain more detailed information on the subcellular localization of the microsomal proteins with cgly, microsomes prepared from Arabidopsis leaf protoplasts were fractionated on isopycnic sucrose gradients (Fig. 5A). An intense peak of anti-cgly reactivity was coincident with the migration of the plasma membrane marker PIP2 (Fig. 5; compare A and C) and a minor peak in very light membrane fractions. In agreement with the results shown in Fig. 4, no peak was detected in the fractions containing the tonoplast marker γ-TIP (Fig. 5; compare A and D). When membranes were treated with Na_2_CO_3_, the general distribution of polypeptides recognized by anti-cgly along the gradient did not change markedly (Fig. 5B). Analysis was also performed by microscopy. The tonoplast can be visualized by immunofluorescence microscopy on fixed and permeabilized plant cells using antisera against specific resident membrane proteins (see, for example, Vera-Estrella *et al.*, 2004; Jia *et al.*, 2013). However, anti-cgly mainly decorated the plasma membrane and in part small circular subcellular structures, but almost no signal was present at the tonoplast (Fig. 6; the red arrows indicate the tonoplast). Subcellular fractionation and microscopy therefore confirmed that the tonoplast is specifically depleted of cgly and indicated that most cgly are at the plasma membrane.

## Discussion

### The Arabidopsis *N*-glycoproteome includes only very few integral membrane proteins of the tonoplast

We have shown here that Arabidopsis *N*-glycosylated membrane proteins with Golgi-modified, complex glycans are almost or totally absent from the tonoplast and are mainly present in the plasma membrane. The tonoplast also has very few glycoproteins with Golgi-unmodified *N*-glycans recognized by ConA. Consistent with this, our *in silico* analysis of tonoplast and plasma membrane proteomes revealed a much lower abundance of potentially *N*-glycosylated polypeptides in the former.

Our biochemistry and bioinformatics results extend, and provide a general explanation for, previous results regarding Arabidopsis *N*-glycosylated proteins. Using ConA affinity chromatography, Minic *et al.* (2007) have identified 102 proteins extracted from Arabidopsis stem. Since they used a protocol mainly suited for the solubilization of hydrolytic enzymes, they detected very few integral membrane proteins, but none of them was assigned to the tonoplast. Arabidopsis *N*-glycopeptides obtained from total protein extracts have also been isolated and analyzed by Zielinska *et al.* (2012) and Song *et al.* (2013), allowing the identification of 1240 and 173 *N*-glycoproteins, respectively. Comparison with our *in silico* data indicate that 23 (TMHMM list) or 24 (Phobius list) PMPs, but no TMPs, are among those identified by Zielinska *et al.* (2012), and six (TMHMM list) or five (Phobius list) PMPs, but again no TMPs, are among those identified by Song *et al.* (2013).

Our results also extend the previous observation that *N*-glycans with the Lewis a epitope are present at the plasma membrane but not at the tonoplast (Fitchette *et al.*, 1999). The Lewis a epitope occurs in a subset of complex glycans: in plants it further elongates the structures that are modified by the addition of β1,2-linked xylose and α1,3-linked fucose, and is also absent from soluble vacuolar proteins. It has been suggested that this may be due to removal by vacuolar hydrolases (Fitchette *et al.*, 1999), in a process similar to the one that removes terminal GlcNAc residues from vacuolar glycoproteins (Vitale and Chrispeels, 1984). Given the fact that, as also shown here, many soluble vacuolar glycoproteins instead contain complex glycans with xylose and fucose, it seems very unlikely that the absence of these residues from tonoplast proteins could be due to their removal by vacuolar hydrolases. It can therefore be concluded that, whereas the absence of glycans with the Lewis a epitope is a common feature of all vacuolar proteins, the scarcity (or total absence) of the entire spectrum of complex glycans is a specific feature of the tonoplast. Plant PMPs traffic through the Golgi complex, and no alternative routes have been identified to date, but both Golgi-dependent and -independent routes to the tonoplast have been identified (Pedrazzini *et al.*, 2013). Furthermore, in meristematic cells, the tonoplast of newly formed vacuoles seems to originate directly from the ER membrane (Marty, 1978; Viotti *et al.*, 2013). Obviously, the glycans of proteins that are transported from the ER to the tonoplast without encountering Golgi enzymes cannot become complex. If a relevant proportion of tonoplast *N*-glycoproteins will be found to follow the Golgi-independent route, this would contribute to explain the scarcity of complex glycans at this membrane, but not the general scarcity of glycans.

### The membrane of inner hydrolytic compartments had divergent evolution

Biochemical analysis was previously performed on *N*-glycosylation in mice (Zielinska *et al.*, 2010). Out of the 7846, 574, and 57 mouse proteins classified, respectively, as ‘intrinsic to membrane’, ‘plasma membrane’ְ, and located at the ‘lysosomal membrane’, 1037 intrinsic to membrane, 123 plasma membrane, and 12 lysosomal membrane proteins were found to be *N*-glycosylated in vivo. These data indicate that the lysosomal membrane is not under-represented within the total mouse membrane *N*-glycoproteome and with respect to the plasma membrane. Although the *in silico* analysis of sequons does not demonstrate actual glycosylation, those numbers are consistent with our analysis showing that there is only a minor difference in the relative proportions of putative *N*-glycosylated proteins between the rat plasma and lysosomal membranes. Therefore, the scarcity of *N*-glycoproteins in the tonoplast has no correspondence in the homologous animal membrane, indicating divergent evolution. When glycopeptides isolated from seven organisms representing the different eukaryotic phyla were compared, most of the divergence was observed in extracellular proteins and PMPs, leading to the conclusion that within the secretory system common cellular functions take place intracellularly whereas specific functions occur in contact with the extracellular environment (Zielinska *et al.*, 2012). Although this may be true as a general observation, our data indicate that the picture can be quite different when more detailed analysis of subcompartments is performed.

Four integral membrane proteins of the LAMP and LIMP families constitute ~50% of the total protein content of the lysosomal membrane and are highly glycosylated with complex glycans (Winchester, 2001). The glycans protect LAMP proteins from *in vivo* proteolysis but are not necessary for LIMP-2 stability (Kundra and Kornfeld, 1999). In LAMP and LIMP proteins, as well as in other LMPs (Schieweck *et al.*, 2009; Gao *et al.*, 2010), most glycans are located in extended luminal terminal domains. Consistently, the only two putative *N*-glycoproteins with more than three sequons that we have assigned to the tonoplast have all six (NAT6), or five out of eight (GLR16), sequons in terminal domains. GLR16 has 87% identity with the glutamate receptor-like channel GLR1.2, which controls calcium fluxes across the plasma membrane in pollen tubes (Michard *et al.*, 2011). Extended glycosylation of the N-terminal domain is common in glutamate receptor-like channels and is involved in receptor function (Dingledine *et al.*, 1999). Many plant integral PMPs have extended domains that are exposed outside the cell and are therefore luminal during synthesis and intracellular trafficking. These domains may have enzymatic properties or recognition functions in signal transduction and are often heavily *N*-glycosylated (Nicol *et al.*, 1998; Jin *et al.*, 2007; Nekrasov *et al.*, 2009), as also indicated by our *in silico* analysis (Supplementary Table S2).

 Glycans may interact with the quality control machinery, favoring correct folding of the long luminal segments, may protect the exposed domains from proteolysis at the cell surface, or may contribute to recognition properties. Glycans that protect from proteolysis or are involved in recognition events are probably exposed on the folded protein surface and therefore have a high probability to be accessible to the action of Golgi enzymes and to become complex. Among the very few putative *N*-glycosylated tonoplast proteins that we have identified, four (cationic amino acid transporter 4, At3g03720.1; ABC transporter B family member 27, At5g39040.1; chloride channel protein CLC-a, At5g40890.1; and vacuolar proton pump subunit a1, At2g28520.1) are functionally related to *N*-glycosylated LMPs. Animal ABCB transporters contain several sequons, hence their original name ‘Permeability-glycoproteins (P-gp)’ (Nielsen *et al.*, 1992). Morover, all mammalian chloride channels are *N*-glycosylated, with the exception of the ClC-7 isoform; this, however, assembles with the highly glycosylated Ostm1 β-subunit (osteopetrosis-associated transmembrane protein 1), proposed to shield ClC-7 from lysosomal degradation (Jentsch, 2008). Our results thus suggest that, at least in ABCB and ClC proteins, *N*-glycosylation could play an important role and has therefore been preserved during evolution.

**Fig. 7.**
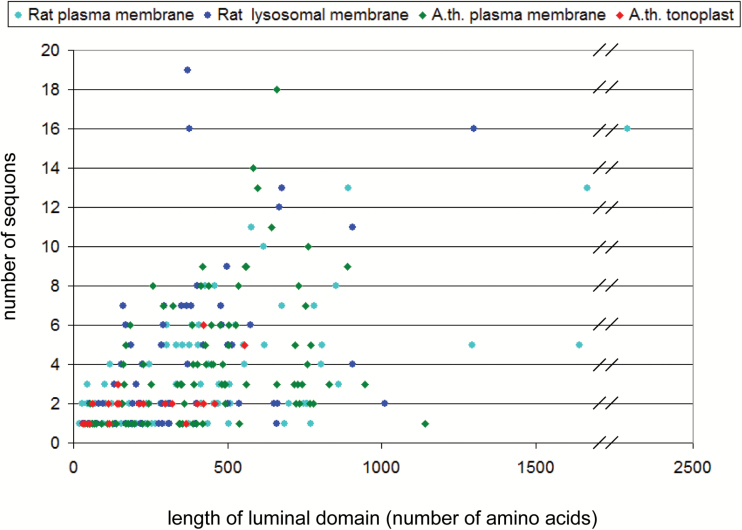
Long luminal domains and multiple sequons are rare in the tonoplast *N*-glycoproteome. The length of luminal domains (*x*-axis) was plotted versus the frequency of sequons (*y*-axis) in the *N*-glycoproteomes of the Arabidopsis tonoplast (red) and plasma membrane (green), and the rat liver lysosomal (blue) and plasma (light blue) membranes.

Finally, it should be underlined that although the four tonoplast polypeptides functionally related to LMPs have potentially glycosylated luminal domains longer than 200 amino acids, extended luminal domains are very infrequent in tonoplast proteins (Fig. 7; Supplementary Table S5), possibly because the main function of the tonoplast is not related to direct interactions with the extracellular environment. Consistently, these four tonoplast proteins and their lysosomal counterparts have at the most three sequons, indicating that in lysosomes these are not among the many membrane proteins with very long, hyperglycosylated luminal domains that characterize the lysosome with respect to the vacuole. This raises the possibility that during evolution protection from unregulated degradation by hydrolytic enzymes could have been achieved in plant cells mainly by limiting the length of luminal domains. A similarly protective function has instead been hypothesized as the major evolutionary pressure for the extensive glycosylation of luminal domains of lysosomal membrane proteins (Schieweck *et al.*, 2009; Gao *et al.*, 2010). This would imply that different evolutionary paths were followed to achieve the same advantage in related subcellular compartments of two different kingdoms.

## Supplementary data

Supplementary data are available at *JXB* online.

Table S1. A.th.TMPs and analysis of their putative *N*-glycosylation sites.

Table S2. A.th.PMPs and analysis of their putative *N*-glycosylation sites.

Table S3. Rat LMPs and analysis of their putative *N*-glycosylation sites.

Table S4. Rat PMPs and analysis of their putative *N*-glycosylation sites.

Table S5. Lists of the lengths of luminal domains and number of identified sequons per domain based on the TMHMM or Phobius software, in *A. thaliana* tonoplast, *A. thaliana* plasma membrane, *R. norvegicus* lysosomal membrane, and *R. norvegicus* plasma membrane proteins.

Supplementary Data
