# Supplementary material for: The Arabidopsis tonoplast is almost devoid of glycoproteins with complex N-glycans, unlike the rat lysosomal membrane
Source: J Exp Bot. 2016 Jan 8;67(6):1769–81. doi: 10.1093/jxb/erv567 (PMC4783361; doi:10.1093/jxb/erv567)
Supplement: Supplementary Data [file supp_67_6_1769__index.html]

The Arabidopsis tonoplast is almost devoid of glycoproteins with complex N-glycans, unlike the rat lysosomal membrane — The Arabidopsis tonoplast is almost devoid of glycoproteins with complex N-glycans, unlike the rat lysosomal membrane — Supplementary Data 

# The Arabidopsis tonoplast is almost devoid of glycoproteins with complex *N*-glycans, unlike the rat lysosomal membrane

## Supplementary Data

Data files

- supplementary\_table\_S1.xls - Supplementary Data
- supplementary\_table\_S2.xls - Supplementary Data
- supplementary\_table\_S3.xls - Supplementary Data
- supplementary\_table\_S4.xls - Supplementary Data
- supplementary\_table\_S5.xls - Supplementary Data
